# Supplementary material for: Arousal state alters brain network switching and moderates cognitive task performance
Source: bioRxiv. 2026 Mar 12:2026.03.09.710652. Preprint. [Version 1] doi: 10.64898/2026.03.09.710652 (PMC13060908; doi:10.64898/2026.03.09.710652)
Supplement: Supplement 1 [file NIHPP2026.03.09.710652v1-supplement-1.pdf]

bioRxiv preprint doi: https://doi.org/10.64898/2026.03.09.710652; this version posted March 12, 2026. The copyright holder for this preprint (which was not certified by peer review) is the author/funder, who has granted bioRxiv a license to display the preprint in perpetuity. It is made available under aCC-BY 4.0 International license.

Supplementary Material

bioRxiv preprint doi: https://doi.org/10.64898/2026.03.09.710652; this version posted March 12, 2026. The copyright holder for this preprint (which was not certified by peer review) is the author/funder, who has granted bioRxiv a license to display the preprint in perpetuity. It is made available under aCC-BY 4.0 International license.

Supplementary Table 1

**HCP-7T**							
Network	***U-value***	***Mean Rank Drowsy***	***Mean Rank Alert***	***z-value***	***r-value***	***p-value***	***q-value***
**ASAL**	6305.00	135.58	181.45	-3.23	-0.17	<0.001	<0.001
**PSAL**	9329.00	185.98	170.88	1.06	0.06	0.28	0.34
**DDMN**	10834.50	211.08	165.62	3.20	0.17	<0.001	<0.001
**VDMN**	10338.00	202.80	167.35	2.50	0.13	0.01	0.02
**LCEN**	8121.00	165.85	175.10	-0.65	-0.04	0.51	0.51
**RCEN**	7542.50	156.21	177.13	-1.47	-0.08	0.14	0.21

**VU-EEG-fMRI**							
Network	***U-value***	***Mean Rank Drowsy***	***Mean Rank Alert***	***z-value***	***r-value***	***p-value***	***q-value***
**ASAL**	73.50	12.63	10.15	0.89	0.19	0.37	0.37
**PSAL**	91.00	14.08	8.40	2.04	0.44	0.04	0.21
**DDMN**	81.00	13.25	9.40	1.38	0.30	0.16	0.33
**VDMN**	44.00	10.17	13.10	-1.06	-0.22	0.29	0.37
**LCEN**	46.00	10.33	12.90	-0.92	-0.20	0.35	0.37
**RCEN**	33.00	9.25	14.20	-1.78	-0.38	0.07	0.21

bioRxiv preprint doi: https://doi.org/10.64898/2026.03.09.710652; this version posted March 12, 2026. The copyright holder for this preprint (which was not certified by peer review) is the author/funder, who has granted bioRxiv a license to display the preprint in perpetuity. It is made available under aCC-BY 4.0 International license.

SAL Parcels	Corresponding Brain Region
a_sal1	Frontal Mid L
a_sal2	Insula L<br>Temporal Pole Sup L
a_sal3	Cingulum Mid Bilateral<br>Supp Motor Area Bilateral<br>Cingulum Ant Bilatera<br>Frontal Sup Bilateral
a_sal4	Frontal Mid R<br>Frontal Sup R
a_sal5	Insula R<br>Frontal Inf Oper R
a_sal6	Cerebellum Crus1 L<br>Cerebellum 6 L
a_sal7	Cerebellum Crus1 R<br>Cerebellum 6 R
p_sal1	Frontal Mid L<br>Frontal Inf Tri L
p_sal2	Supramarginal L<br>Parietal Inf L<br>Angular L
p_sal3	Precuneus L
p_sal4	Cingulum Mid R
p_sal5	Parietal Sup R<br>Postcentral R<br>Precuneus R
p_sal6	Supramarginal R<br>Parietal Inf R<br>Rolandic Oper R<br>Temporal Sup R
p_sal7	Thalamus L<br>Hippocampus L
p_sal8	Cerebellum 8 L<br>Cerebellum 6 L<br>Cerebellum_4_5 L<br>Cerebellum 7b L<br>Cerebellum Crus1 L
p_sal9	Insula L<br>Putamen L
p_sal10	Thalamus R
	Cerebellum 8 R

DMN parcels	Corresponding Brain Region
d_dmn1	Cingulum Ant Bilateral<br>Rectus Bilateral<br>Frontal Mid Orb Bilateral<br>Frontal Sup Medial Bilateral<br>Frontal Sup L
d_dmn2	Angular L
d_dmn3	Frontal Sup R
d_dmn4	Precuneus Bilateral<br>Cingulam Post Bilateral<br>Calcarine Bilateral
d_dmn5	Cingulam Mid Bilateral
d_dmn6	Angular R
d_dmn7	Thalamus Bilateral
d_dmn8	Para Hippocampal L<br>Fusiform L<br>Hippocampus L
d_dmn9	Para Hippocampal R
v_dmn1	Calcarine L<br>Lingual L<br>Precuneus L<br>Cuneus L
v_dmn2	Frontal Mid L<br>Frontal Sup L
v_dmn3	Fusiform L
v_dmn4	Occipital Mid L
v_dmn5	Precuneus R<br>Calcarine R<br>Lingual R
v_dmn6	Precuneus (Bilateral)
v_dmn7	Frontal Sup R<br>Frontal Mid R
v_dmn8	Fusiform R
v_dmn9	Occipital Mid R<br>Temporal Mid R<br>Angular R
v_dmn10	Cerebellum 9 R<br>Cerebellum 8 R

CEN parcels	Corresponding Brain Region
lcen_1	Frontal Mid L<br>Frontal Sup Medial L<br>Frontal Inf Oper L<br>Precentral L<br>Frontal Sup L<br>Frontal Inf Tri L
lcen_2	Frontal Inf Tri L<br>Frontal Inf Orb L<br>Frontal Mid Orb L<br>Frontal Sup L
lcen_3	Parietal Inf L<br>Angular L<br>Occipital Mid L<br>Parietal Sup L
lcen_4	Temporal Mid L<br>Temporal Inf L
lcen_5	Cerebellum Crus2 R<br>Cerebellum Crus1 R<br>Cerebellum 7b L<br>Cerebellum Crus1 R
lcen_6	Thalamus L
rcen_1	Frontal Mid R<br>Frontal Sup R<br>Frontal Inf Oper R<br>Front Inf Tri R
rcen_2	Frontal Mid R<br>Frontal Mid Orb R<br>Frontal Inf Orb R
rcen_3	Angular R<br>Parietal Inf R<br>Supramarginal R
rcen_4	Frontal Sup Medial R
rcen_5	Cerebellum Crus2 L<br>Cerebellum 8 L<br>Cerebellum 7b L
rcen_6	Caudate R<br>Thalamus R

bioRxiv preprint doi: https://doi.org/10.64898/2026.03.09.710652; this version posted March 12, 2026. The copyright holder for this preprint (which was not certified by peer review) is the author/funder, who has granted bioRxiv a license to display the preprint in perpetuity. It is made available under aCC-BY 4.0 International license.

**p_sal11**	Cerebellum Crus1 R<br>Cerebellum Crus2 R
**p_sal12**	Insula R

**HCP-7T**							
**Parcel**	***U-value***	***Mean Rank Drowsy***	***Mean Rank Alert***	***z-value***	***r-value***	***p-value***	***q-value***
**d_dmn1**	11147.50	216.29	164.52	3.64	0.20	<0.001	<0.001
**d_dmn2**	10596.50	207.11	166.45	2.86	0.15	<0.001	0.01
**d_dmn3**	10006.00	197.27	168.51	2.02	0.11	0.04	0.09
**d_dmn4**	12296.50	235.44	160.51	5.28	0.28	<0.001	<0.001
**d_dmn5**	7857.50	161.46	176.03	-1.03	-0.06	0.30	0.40
**d_dmn6**	11279.50	218.49	164.06	3.83	0.21	<0.001	<0.001
**d_dmn7**	4179.50	100.16	188.89	-6.25	-0.34	<0.001	<0.001
**d_dmn8**	9643.50	191.23	169.78	1.51	0.08	0.13	0.22
**d_dmn9**	8483.00	171.88	173.84	-0.14	-0.01	0.89	0.89
**v_dmn1**	10139.00	199.48	168.05	2.21	0.12	0.03	0.06
**v_dmn2**	9556.00	189.77	170.09	1.39	0.07	0.17	0.25
**v_dmn3**	9042.00	181.20	171.88	0.66	0.04	0.51	0.59
**v_dmn4**	9773.00	193.38	169.33	1.69	0.09	0.09	0.16
**v_dmn5**	10313.50	202.39	167.44	2.46	0.13	0.01	0.03
**v_dmn6**	9613.00	190.72	169.89	1.47	0.08	0.14	0.23
**v_dmn7**	11075.50	215.09	164.77	3.54	0.19	<0.001	<0.001
**v_dmn8**	8145.00	166.25	175.02	-0.62	-0.03	0.54	0.61

bioRxiv preprint doi: https://doi.org/10.64898/2026.03.09.710652; this version posted March 12, 2026. The copyright holder for this preprint (which was not certified by peer review) is the author/funder, who has granted bioRxiv a license to display the preprint in perpetuity. It is made available under aCC-BY 4.0 International license.

**v_dmn9**	9324.00	185.90	170.90	1.06	0.06	0.29	0.40
**v_dmn10**	8981.50	180.19	172.10	0.57	0.03	0.57	0.63
**a_sal1**	7354.00	153.07	177.79	-1.74	-0.09	0.08	0.15
**a_sal2**	9747.00	192.95	169.42	1.66	0.09	0.10	0.17
**a_sal3**	7710.00	159.00	176.54	-1.24	-0.07	0.22	0.32
**a_sal4**	7257.50	151.46	178.12	-1.88	-0.10	0.06	0.12
**a_sal5**	10864.50	211.58	165.51	3.24	0.17	<0.001	<0.001
**a_sal6**	4413.50	104.06	188.07	-5.91	-0.32	<0.001	<0.001
**a_sal7**	4556.50	106.44	187.57	-5.71	-0.31	<0.001	<0.001
**p_sal1**	8861.50	178.19	172.52	0.40	0.02	0.69	0.75
**p_sal2**	9092.00	182.03	171.71	0.73	0.04	0.47	0.57
**p_sal3**	10722.00	209.20	166.01	3.04	0.16	<0.001	0.01
**p_sal4**	9168.50	183.31	171.44	0.84	0.04	0.40	0.50
**p_sal5**	10591.50	207.03	166.47	2.86	0.15	<0.001	0.01
**p_sal6**	9345.00	186.25	170.83	1.09	0.06	0.28	0.39
**p_sal7**	6145.00	132.92	182.01	-3.46	-0.19	<0.001	<0.001
**p_sal8**	5161.00	116.52	185.45	-4.85	-0.26	<0.001	<0.001
**p_sal9**	10334.00	202.73	167.37	2.49	0.13	0.01	0.03
**p_sal10**	4757.50	109.79	186.87	-5.43	-0.29	<0.001	<0.001
**p_sal11**	5129.50	115.99	185.56	-4.90	-0.26	<0.001	<0.001
**p_sal12**	10765.50	209.93	165.86	3.10	0.17	<0.001	0.01
**lcen_1**	10598.50	207.14	166.44	2.87	0.15	<0.001	0.01
**lcen_2**	8348.50	169.64	174.31	-0.33	-0.02	0.74	0.79
**lcen_3**	9586.00	190.27	169.98	1.43	0.08	0.15	0.24

bioRxiv preprint doi: https://doi.org/10.64898/2026.03.09.710652; this version posted March 12, 2026. The copyright holder for this preprint (which was not certified by peer review) is the author/funder, who has granted bioRxiv a license to display the preprint in perpetuity. It is made available under aCC-BY 4.0 International license.

**lcen_4**	9062.00	181.53	171.81	0.68	0.04	0.49	0.59
**lcen_5**	6284.00	135.23	181.53	-3.26	-0.18	<0.001	<0.001
**lcen_6**	6657.00	141.45	180.22	-2.73	-0.15	0.01	0.01
**rcen_1**	8682.50	175.21	173.14	0.15	0.01	0.88	0.89
**rcen_2**	7939.00	162.82	175.74	-0.91	-0.05	0.36	0.46
**rcen_3**	7862.00	161.53	176.01	-1.02	-0.05	0.31	0.40
**rcen_4**	8713.00	175.72	173.03	0.19	0.01	0.85	0.89
**rcen_5**	5551.50	123.03	184.09	-4.30	-0.23	<0.001	<0.001
**rcen_6**	3593.00	90.38	190.94	-7.08	-0.38	<0.001	<0.001

**VU-EEG-fMRI**							
**Parcel**	***U-value***	***Mean Rank Drowsy***	***Mean Rank Alert***	***z-value***	***r-value***	***p-value***	***q-value***
**d_dmn_1**	84.00	13.50	9.10	1.58	0.34	0.11	0.27
**d_dmn_2**	78.00	13.00	9.70	1.19	0.25	0.23	0.40
**d_dmn_3**	71.50	12.46	10.35	0.76	0.16	0.45	0.60
**d_dmn_4**	86.00	13.67	8.90	1.71	0.37	0.09	0.25
**d_dmn_5**	48.00	10.50	12.70	-0.79	-0.17	0.43	0.59
**d_dmn_6**	62.00	11.67	11.30	0.13	0.03	0.89	0.93
**d_dmn_7**	6.50	7.04	16.85	-3.53	-0.75	0.00	0.02
**d_dmn_8**	50.00	10.67	12.50	-0.66	-0.14	0.51	0.65

bioRxiv preprint doi: https://doi.org/10.64898/2026.03.09.710652; this version posted March 12, 2026. The copyright holder for this preprint (which was not certified by peer review) is the author/funder, who has granted bioRxiv a license to display the preprint in perpetuity. It is made available under aCC-BY 4.0 International license.

**d_dmn_9**	61.00	11.58	11.40	0.07	0.01	0.95	0.97
**v_dmn_1**	74.00	12.67	10.10	0.92	0.20	0.35	0.55
**v_dmn_2**	82.00	13.33	9.30	1.45	0.31	0.15	0.33
**v_dmn_3**	56.50	11.21	11.85	-0.23	-0.05	0.82	0.87
**v_dmn_4**	68.00	12.17	10.70	0.53	0.11	0.60	0.74
**v_dmn_5**	76.00	12.83	9.90	1.06	0.22	0.29	0.48
**v_dmn_6**	47.00	10.42	12.80	-0.86	-0.18	0.39	0.57
**v_dmn_7**	94.50	14.38	8.05	2.27	0.49	0.02	0.16
**v_dmn_8**	52.50	10.88	12.25	-0.49	-0.11	0.62	0.74
**v_dmn_9**	85.50	13.63	8.95	1.68	0.36	0.09	0.25
**v_dmn_10**	41.50	9.96	13.35	-1.22	-0.26	0.19	0.38
**a_sal_1**	53.50	10.96	12.15	-0.43	-0.09	0.67	0.76
**a_sal_2**	66.50	12.04	10.85	0.43	0.09	0.67	0.76
**a_sal_3**	34.50	9.38	14.05	-1.68	-0.36	0.09	0.25
**a_sal_4**	41.00	9.92	13.40	-1.25	-0.27	0.21	0.40
**a_sal_5**	71.00	12.42	10.40	0.73	0.15	0.47	0.61
**a_sal_6**	25.00	8.58	15.00	-2.31	-0.49	0.02	0.16
**a_sal_7**	29.00	8.92	14.60	-2.04	-0.44	0.04	0.22
**p_sal_1**	104.50	15.21	7.05	2.93	0.63	0.00	0.05
**p_sal_2**	88.50	13.88	8.65	1.88	0.40	0.06	0.23
**p_sal_3**	47.00	10.42	12.80	-0.86	-0.18	0.39	0.57
**p_sal_4**	80.50	13.21	9.45	1.35	0.29	0.17	0.37
**p_sal_5**	78.50	13.04	9.65	1.22	0.26	0.22	0.40
**p_sal_6**	93.50	14.29	8.15	2.21	0.47	0.03	0.16

bioRxiv preprint doi: https://doi.org/10.64898/2026.03.09.710652; this version posted March 12, 2026. The copyright holder for this preprint (which was not certified by peer review) is the author/funder, who has granted bioRxiv a license to display the preprint in perpetuity. It is made available under aCC-BY 4.0 International license.

**p_sal_7**	17.50	7.96	15.75	-2.80	-0.60	0.00	0.06
**p_sal_8**	32.50	9.21	14.25	-1.81	-0.39	0.07	0.25
**p_sal_9**	85.00	13.58	9.00	1.65	0.35	0.10	0.26
**p_sal_10**	25.50	8.63	14.95	-2.27	-0.49	0.02	0.16
**p_sal_11**	33.00	9.25	14.20	-1.78	-0.38	0.07	0.25
**p_sal_12**	90.50	14.04	8.45	2.01	0.43	0.04	0.22
**lcen_1**	60.50	11.54	11.45	0.03	0.01	0.97	0.97
**lcen_2**	67.50	12.13	10.75	0.49	0.11	0.62	0.74
**lcen_3**	55.00	11.08	12.00	-0.33	-0.07	0.74	0.82
**lcen_4**	84.50	13.54	9.05	1.62	0.34	0.10	0.26
**lcen_5**	42.00	10.00	13.30	-1.19	-0.25	0.23	0.40
**lcen_6**	31.00	9.08	14.40	-1.91	-0.41	0.05	0.23
**rcen_1**	30.50	9.04	14.45	-1.95	-0.41	0.05	0.23
**rcen_2**	63.50	11.79	11.15	0.23	0.05	0.82	0.87
**rcen_3**	40.00	9.83	13.50	-1.32	-0.28	0.18	0.38
**rcen_4**	46.00	10.33	12.90	-0.92	-0.20	0.35	0.55
**rcen_5**	48.00	10.50	12.70	-0.79	-0.17	0.43	0.59
**rcen_6**	11.50	7.46	16.35	-3.20	-0.68	0.00	0.03

Supplementary Table 1. Statistical testing of arousal-dependent changes in network flexibility. Mann-Whitney U test statistics for network flexibility analyses with (a) sub-networks as nodes, or (b) label of parcels as nodes (c) statistical results for HCP-7T parcel level, (d) statistical results for VU-EEG-fMRI parcel level.

bioRxiv preprint doi: https://doi.org/10.64898/2026.03.09.710652; this version posted March 12, 2026. The copyright holder for this preprint (which was not certified by peer review) is the author/funder, who has granted bioRxiv a license to display the preprint in perpetuity. It is made available under aCC-BY 4.0 International license.

**Data: VU EEG-fMRI**					**Data: HCP-7T**	
**Networks**	**Null 1** (temporal & spatial mean)	**Null 2** (Null 1 + spatial & temporal variance)	**Null 3** (Null 2 + global signal correls)	**Null 4** (Null 3 + static correlations)	**Null 3** (Null 2 + global signal correls)	**Null 4** (Null 3 + static correlations)
**PSAL**	0.53	0.55	0.59	0.66	0.2	1
**ASAL**	0.88	0.92	0.9	0.9	<0.001	0.4
**LCEN**	0.3	0.28	0.32	0.52	0.9	0.7
**RCEN**	0.31	0.22	0.37	0.48	1	0.2
**DDMN**	<0.001	<0.001	0.02	0.13	<0.001	1
**VDMN**	0.88	0.78	0.86	1	<0.001	1

bioRxiv preprint doi: https://doi.org/10.64898/2026.03.09.710652; this version posted March 12, 2026. The copyright holder for this preprint (which was not certified by peer review) is the author/funder, who has granted bioRxiv a license to display the preprint in perpetuity. It is made available under aCC-BY 4.0 International license.

VU-EEG-fMRI					HCP-7T	
**Parcels**	**Null 1**	**Null 2**	**Null 3**	**Null 4**	**Null 3**	**Null 4**
**a_sal1**	0.3	0.47	0.48	0.28	0.1	1
**a_sal2**	0.56	0.69	0.6	0.66	0.3	1
**a_sal3**	0.63	0.69	0.61	0.55	1	1
**a_sal4**	0.44	0.59	0.6	0.46	0.5	1
**a_sal5**	0.93	0.92	0.97	0.92	<0.001	0.9
**a_sal6**	0.06	0.15	0.12	0.07	<0.001	<0.001
**a_sal7**	0.73	0.84	0.81	0.85	<0.001	0.1
**p_sal1**	0.05	0.05	0.05	0.03	0.8	1
**p_sal2**	0.53	0.43	0.45	0.54	0.4	1
**p_sal3**	0.54	0.72	0.71	0.62	0.1	0.8
**p_sal4**	0.23	0.2	0.36	0.19	0.8	1
**p_sal5**	0.62	0.62	0.62	0.61	<0.001	1
**p_sal6**	0.22	0.36	0.3	0.09	0.7	1
**p_sal7**	<0.001	<0.001	0.01	<0.001	0.1	1
**p_sal8**	0.23	0.36	0.34	0.32	<0.001	0.2
**p_sal9**	0.42	0.63	0.59	0.54	0.4	1
**p_sal10**	<0.001	<0.001	<0.001	<0.001	<0.001	0.2
**p_sal11**	0.45	0.51	0.56	0.5	<0.001	0.3
**p_sal12**	0.13	0.2	0.21	0.04	0.2	0.8
**lcen_1**	0.63	0.72	0.67	0.72	0.1	0.9
**lcen_2**	0.77	0.82	0.9	0.85	0.8	1

bioRxiv preprint doi: https://doi.org/10.64898/2026.03.09.710652; this version posted March 12, 2026. The copyright holder for this preprint (which was not certified by peer review) is the author/funder, who has granted bioRxiv a license to display the preprint in perpetuity. It is made available under aCC-BY 4.0 International license.

**lcen_3**	0.6	0.76	0.78	0.79	0.6	1
**lcen_4**	0.75	0.86	0.83	0.86	0.7	1
**lcen_5**	0.41	0.47	0.58	0.41	0.2	0.9
**lcen_6**	<0.001	0.01	0.04	<0.001	<0.001	1
**rcen_1**	0.98	1	1	0.97	1	1
**rcen_2**	0.76	0.74	0.76	0.68	0.6	1
**rcen_3**	0.98	1	0.97	0.99	0.7	1
**rcen_4**	0.59	0.65	0.74	0.58	0.8	1
**rcen_5**	0.11	0.15	0.17	0.05	<0.001	0.5
**rcen_6**	<0.001	<0.001	0.01	<0.001	<0.001	<0.001
**d_dmn1**	0.2	0.19	0.33	0.13	<0.001	0.7
**d_dmn2**	0.29	0.38	0.41	0.29	0.2	1
**d_dmn3**	0.89	0.91	0.93	0.98	0.6	1
**d_dmn4**	0.24	0.25	0.32	0.2	<0.001	<0.001
**d_dmn5**	0.42	0.5	0.61	0.44	0.9	1
**d_dmn6**	0.99	1	1	0.98	0.1	0.5
**d_dmn7**	<0.001	<0.001	<0.001	<0.001	<0.001	0.2
**d_dmn8**	0.57	0.65	0.68	0.64	0.5	1
**d_dmn9**	0.52	0.6	0.67	0.71	0.9	1
**v_dmn1**	0.13	0.28	0.23	0.11	0.5	1
**v_dmn2**	0.61	0.74	0.73	0.64	0.1	1
**v_dmn3**	0.45	0.58	0.57	0.49	0.9	1
**v_dmn4**	0.73	0.85	0.83	0.84	0.4	1

bioRxiv preprint doi: https://doi.org/10.64898/2026.03.09.710652; this version posted March 12, 2026. The copyright holder for this preprint (which was not certified by peer review) is the author/funder, who has granted bioRxiv a license to display the preprint in perpetuity. It is made available under aCC-BY 4.0 International license.

**v_dmn5**	0.22	0.32	0.41	0.27	0.1	0.9
**v_dmn6**	0.47	0.59	0.5	0.47	0.2	1
**v_dmn7**	0.31	0.3	0.4	0.31	<0.001	0.9
**v_dmn8**	0.3	0.44	0.49	0.4	0.9	1
**v_dmn9**	0.19	0.31	0.33	0.17	0.5	1
**v_dmn10**	0.01	0.1	0.06	<0.001	0.9	1

Supplementary Table 2. Testing which BOLD signal characteristics contribute to arousal-dependent network flexibility. a) Null model results for network-level analysis. b) Null model results for parcel-level analysis. For parcel level labels of brain regions please refer to Supplementary Table1b. Null models progressively maintained the following characteristics of the fMRI data: (1) mean across time and across nodes, (2) variation across time and across nodes, (3) the correlation between each node and the global signal, and lastly, (4) static correlation between salience, default and central executive subnetworks.

bioRxiv preprint doi: https://doi.org/10.64898/2026.03.09.710652; this version posted March 12, 2026. The copyright holder for this preprint (which was not certified by peer review) is the author/funder, who has granted bioRxiv a license to display the preprint in perpetuity. It is made available under aCC-BY 4.0 International license.

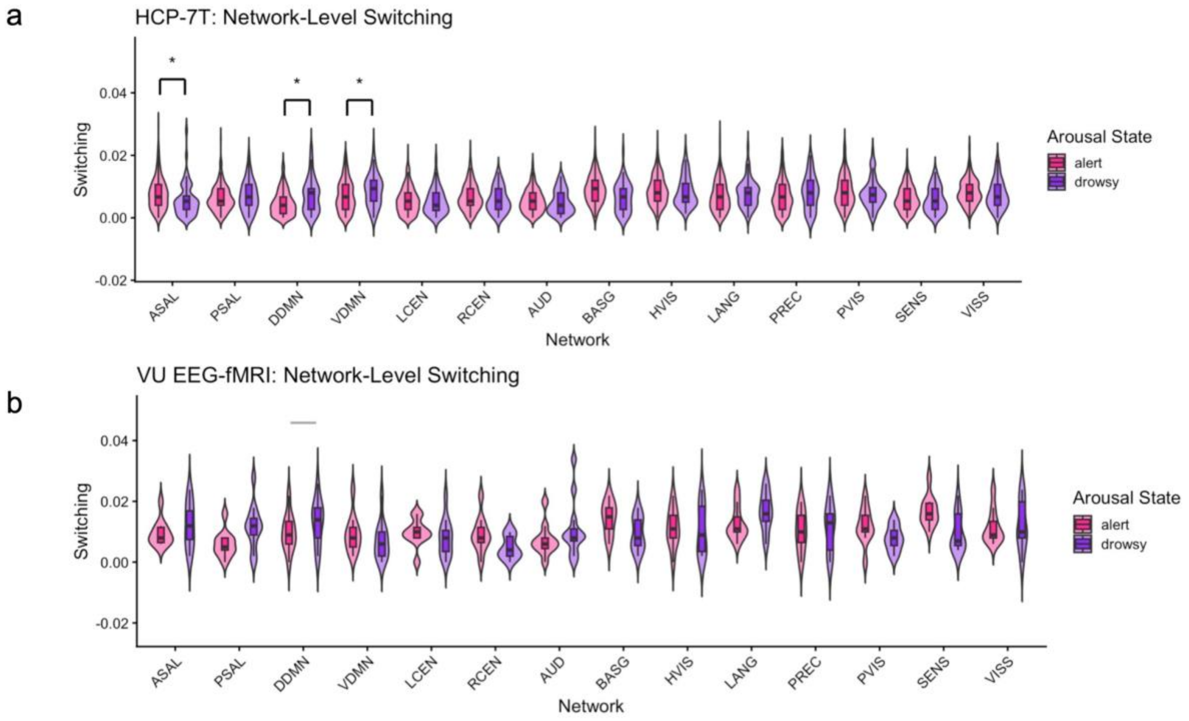

Supplementary Figure 1. Extended network switching across arousal state. a) HCP-7T data: Violin plots show network-level switching across all 14 large-scale networks in the FINDLAB atlas for alert (pink) and drowsy (purple) arousal states. Auditory Network (AUD), Basil Ganglia Network (BASG), Higher Visual Network (HVIS), language network (LANG), precuneus network (PREC), Visual Network I (PVIS), Sensorimotor Network (SENS), Visual Network II (VISS) Note: AUD, BASG, HVIS, LANG, PREC, PVIS, SENS, VISS networks shown here were not tested for significant differences. b) Similar plot but for VU-EEG-fMRI dataset, the grey bar signifies this network was significant up to the third null model.

bioRxiv preprint doi: https://doi.org/10.64898/2026.03.09.710652; this version posted March 12, 2026. The copyright holder for this preprint (which was not certified by peer review) is the author/funder, who has granted bioRxiv a license to display the preprint in perpetuity. It is made available under aCC-BY 4.0 International license.

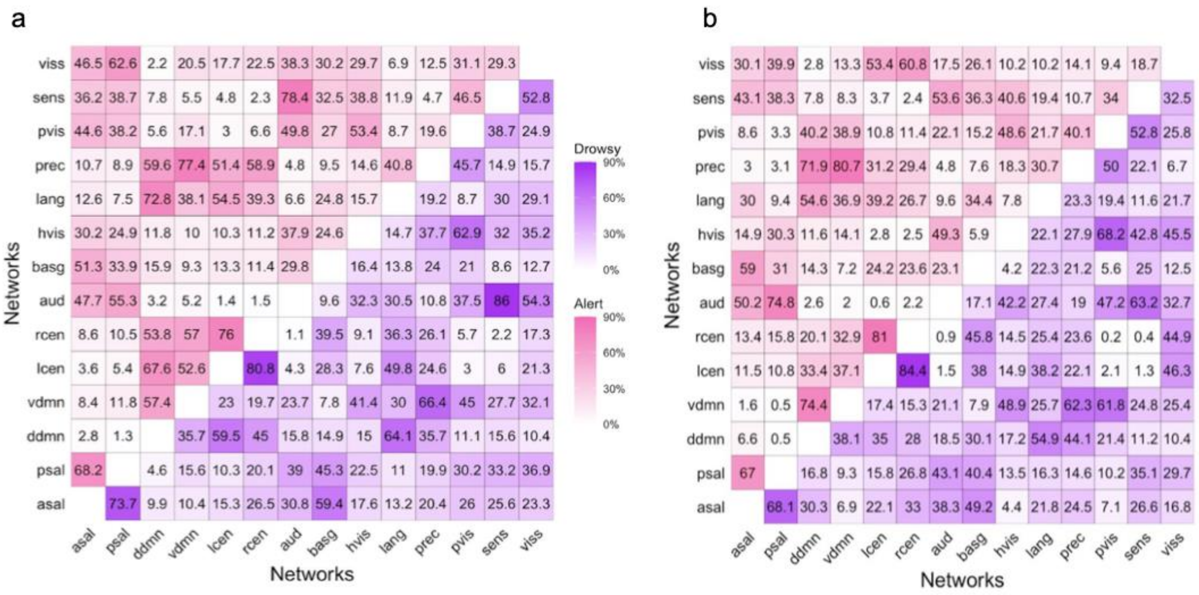

Supplementary Figure 2. State-dependent community allegiance of all subnetworks in the FINDLAB atlas, shown for a) the HCP-7T dataset and b) the VU-EEG-fMRI dataset.
